# Growth and human milk intake outcomes from postdischarge feedings among very‐low‐birth‐weight preterm infants: A systematic review

**DOI:** 10.1002/ncp.70127

**Published:** 2026-04-29

**Authors:** Tara Rebele, Allison Gomes, Melanie Newkirk, Mina Ghajar, Laura Byham‐Gray, Jane Ziegler

**Affiliations:** ^1^ Department of Clinical and Preventive Nutrition Sciences, Rutgers The State University of New Jersey New Brunswick New Jersey USA

**Keywords:** breastfeeding, growth, human milk fortification, postdischarge formula, postdischarge nutrition, preterm infant

## Abstract

**Background:**

Preterm infants of <34 weeks gestation or <1500 g are at risk for postnatal growth failure and often require nutrition support after discharge. Despite expert recommendations for postdischarge human milk (HM) fortification or nutrient‐enriched formula feeding, the effects of current feeding strategies on growth and HM intake remain unclear. This systematic review evaluated the impact of postdischarge fortified expressed HM or direct breastfeeding (BF) with postdischarge formula (PDF) bottle feeds compared to unfortified HM or BF with standard term formula bottle feeds on growth and HM intake outcomes through 6 and 12 months corrected age (CA).

**Methods:**

A systematic search of eight databases and grey literature sources was conducted for studies published in English since 2012 identifying 3146 studies. Risk of bias was assessed using standardized Cochrane RoB 2 and ROBINS‐I tools. Meta‐analysis was not conducted due to heterogeneity in interventions, outcome timing, and outcome metrics; results were narratively synthesized.

**Results:**

Four studies (*n* = 2 randomized controlled trials, *n* = 2 cohorts), including 548 preterm infants, met eligibility criteria. Two studies showed significantly greater weight gain, and one showed significantly greater head circumference in the fortified groups at 6 months CA compared to the unfortified groups. Length differences were not significant. Three studies reporting HM intake found comparable or greater HM intake in the fortified groups.

**Conclusion:**

Current evidence suggests that postdischarge fortified HM or direct BF with supplemental PDF bottle feeds may support improved weight and head circumference outcomes without compromising HM intake. However, methodological variability highlights the need for standardized, prospective studies.

## INTRODUCTION

Preterm infants born <34 weeks gestational age or weighing <1500 g are at high risk for nutrient deficits and postnatal growth failure.^1^, ^2^ Despite advances in neonatal nutrition aimed at meeting estimated energy, protein, and micronutrient needs, growth outcomes often remain suboptimal.^3^ In the United States, more than half of preterm infants are discharged from the neonatal intensive care unit (NICU) with postnatal growth failure.^3^, ^4^, ^5^, ^6^ Compared to term infants, preterm infants have greater energy, protein, and micronutrient requirements to support adequate growth (Table 1).^8^, ^9^ These increased needs persist beyond discharge and may necessitate ongoing nutrition support to promote catch‐up growth, particularly in infants with greater prematurity, accumulated nutrient deficits, or growth failure.^3^, ^6^, ^10^

**Table 1:** Preterm and term infant estimated energy and protein needs.

GA (weeks)	Energy (kcal/kg/day)	Protein (g/kg/day)
Preterm infant estimated nutrition needs by GA^3^		
<28	125	4.0
28–31	125	3.9
32–33	130	3.5
37–38	115	2.5
Healthy term infant DRI for energy and protein^7^		
	~106	1.5

Abbreviations: DRI, Dietary Reference Intake; GA, gestational age.

Human milk (HM) is preferred over infant formula due to its immunologic and bioactive properties, which help protect against comorbidities such as necrotizing enterocolitis, chronic lung disease, and cognitive impairment.^3^, ^11^, ^12^, ^13^ However, the nutrient content of unfortified HM is insufficient to meet the increased energy, protein, calcium, phosphorus, sodium, and vitamin D requirements of preterm infants.^9^, ^10^ To address these deficits and support optimal growth in the NICU, expressed HM is routinely fortified with commercial multi‐nutrient human milk fortifiers (HMFs)—products that increase the energy, protein and micronutrient content of HM.^9^ With improved lactation support and an increased emphasis on HM‐based diets in the NICU, HM intake at discharge among preterm infants in the United States rose from 44% to 52% between 2008 and 2017.^14^ Although HM remains the preferred feeding choice after discharge,^9^ unfortified HM may be insufficient to correct accumulated nutrition deficits or support catch‐up growth.^15^ To bridge this gap, expert consensus guidelines recommend continued nutrition support through fortified expressed HM feedings or nutrient‐enriched postdischarge formula (PDF).^3^, ^6^, ^9^, ^10^, ^15^, ^16^, ^17^ PDF is a nutrient‐enriched infant formula specifically designed to support growth in preterm or low‐birthweight (LBW) infants after discharge.^8^, ^18^

While fortified HM is recommended post discharge, consensus is lacking on both the optimal postdischarge fortification strategy and how best to supplement the directly breastfed (BF) infant.^3^, ^8^, ^15^, ^17^ As a result, several feeding strategies have emerged in clinical practice, including fortified expressed HM and supplemental formula bottle feeds (Figure 1).^3^, ^6^, ^9^, ^15^, ^16^, ^17^, ^19^ Because commercial HMFs are generally only available in NICUs, powder PDFs—readily accessible in retail settings—are often used after discharge and mixed with expressed HM for nutrient enrichment in place of commercial HMF. For the directly BF infant, PDF may be provided as several daily supplemental bottle feeds.^6^, ^16^, ^19^ As shown in Table 2, fortified HM and PDF at 22–24 kcal/oz provide 10–20% more energy and 40%–58% more protein than unfortified HM and standard term formula.^8^, ^14^ Additional calcium and phosphorus are also essential for preterm infants to support bone mineralization.^3^ While not the focus of this review, it is important to note that fortified HM provides three to four times more calcium and phosphorus than unfortified HM, and PDFs provide 50%–60% more than standard term formula.^8^, ^14^

**Figure 1:**
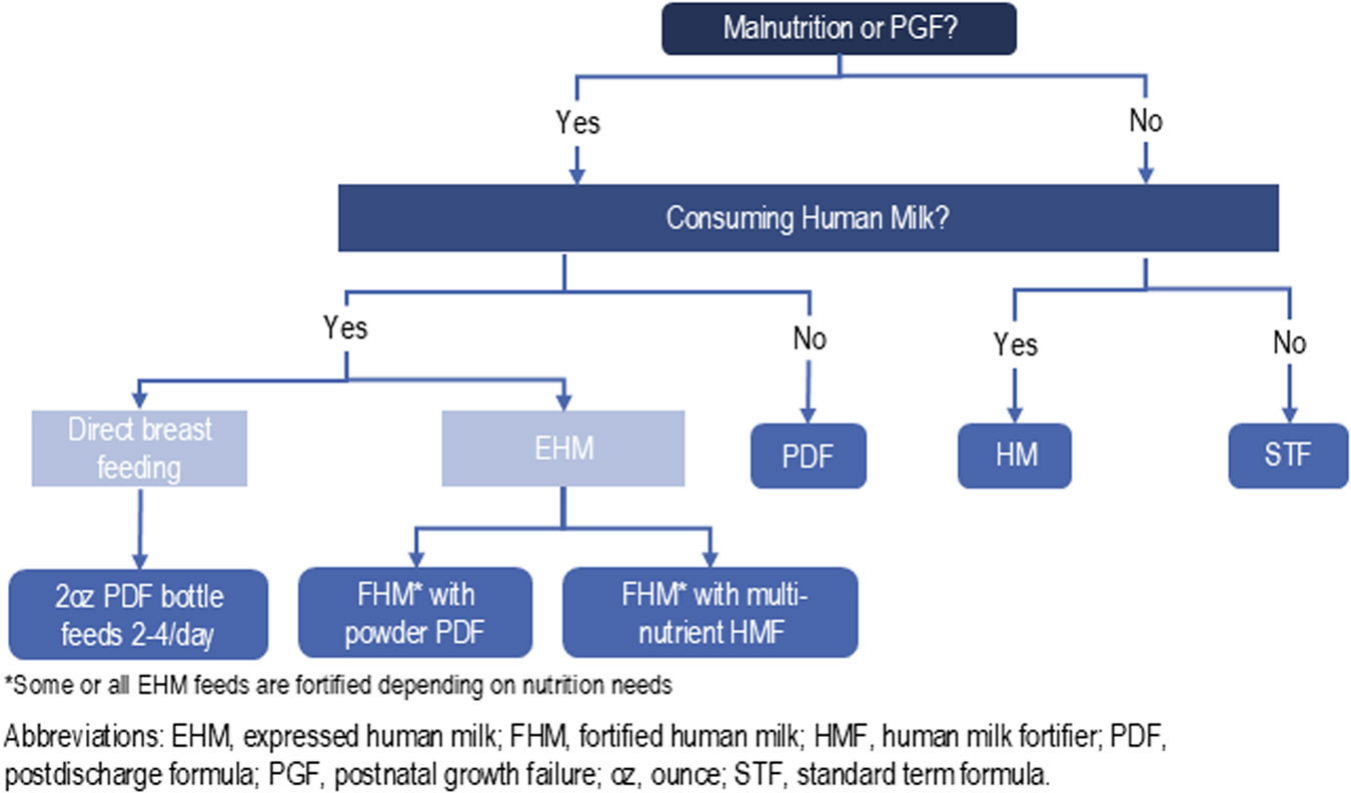
Flow chart of postdischarge nutrition options for preterm infants.^3^, ^6^, ^9^, ^15^, ^16^, ^17^, ^19^

**Table 2:** Typical macronutrient content of common postdischarge feeding options.^15^, ^20^

Per 100 mL	HM	STF	PTF	PDF	EHM + HMF^a^	EHM + PDF^b^	HM + B‐PDF^c^
Energy (kcal)	67	67	80–100	74	74–80	74–80	68–71
Protein (g)	1.3	1.4–1.5	2–3.3	2–2.1	2–2.8	1.4–1.5	1.6–1.7
Fat (g)	5.2	3.6‐3.7	3.4‐6.7	3.9–4.1	2.9–5	3.9–4.2	4.5–4.6

Abbreviations: EHM, expressed human milk; HM, human milk; PDF, postdischarge formula; PTF, preterm formula; STF, standard term formula.

^a^ EHM + HMF: fortified with commercially available multi‐nutrient bovine powder and/or liquid human milk fortifier mixed to 22–24 kcal/oz.

^b^ EHM + PDF: fortified with powder PDF mixed to 22–24 kcal/oz.

^c^ HM + B‐PDF: HM feeding supplemented with 2–4 2‐oz bottles of PDF.

Although expert consensus recommends postdischarge fortification of expressed HM or the use of nutrient‐enriched PDF for preterm infants with postnatal growth failure, current evidence does not clearly support these feeding practices.^21^ A 2013 Cochrane review (*n* = 2 studies, *n* = 246 preterm infants) found that multi‐nutrient HM fortification for 3–4 months post discharge had no significant effect on achieved growth in weight (g), length (cm), or head circumference (HC) (cm).^21^ Postdischarge HM fortification or direct BF with PDF bottles are intended to meet a preterm infant's higher nutrition needs without reducing the volume and duration of HM intake.^8^, ^9^ However, the perception that infant formulas, including PDFs, are superior may lead to decreased HM intake and increased formula consumption after discharge.^21^ Additionally, a study by Erikson et al. reported reduced breastfeeding continuation after NICU discharge. However, the impact of HM fortification or supplemental PDF bottle feeds on breastfeeding rates were not evaluated.^22^ Furthermore, discharge nutrition practices have evolved since the 2013 Cochrane review, and the impact of current strategies, such as PDF‐fortified expressed HM and direct BF with PDF bottle feeds, on growth and HM intake outcomes through 12 months corrected age (CA) remain unclear.^18^, ^21^, ^23^ This knowledge gap highlights the need for updated, evidence‐based guidance on postdischarge feeding strategies, including PDF‐fortified HM and direct BF with PDF bottle feeds.^3^, ^6^, ^15^, ^16^, ^17^

### Objectives

The objective of this systematic review was to compare the effects of current postdischarge feeding strategies on growth and HM intake from NICU discharge through 6 and 12 months corrected age (CA) among preterm infants born <34 weeks gestational age or <1500 g. Feeding strategies included fortified expressed HM (multi‐nutrient HMF or powder PDF) and/or direct BF with supplemental PDF bottle feeds, compared to unfortified HM feedings and/or direct BF with supplemental standard term formula bottle feeds. We hypothesized that preterm infants receiving fortified expressed HM or directly BF with supplemental PDF bottle feeds would have (1) improved growth outcomes from discharge through 6 and 12 months CA, and (2) sustained HM intake outcomes from discharge through 6 and 12 months CA.

## METHODS

### Study overview

The *Cochrane Handbook on Systematic Reviews for Interventions* was used to develop the methodology of this systematic review, encompassing defined objectives, clear inclusion criteria for studies, reproducible methodology, a systematic approach to the literature search, risk of bias assessment, and synthesis of the extracted data.^24^ In addition, the Preferred Reporting Items for Systematic Reviews and Meta‐analyses (PRISMA) was used in reporting the methods, inclusive of the PRISMA flow diagram of the literature search results and study selection process, and in reporting the results of this systematic review.^25^ PRISMA checklists and study variables are detailed in Tables S1–S3.

### Eligibility criteria

Study inclusion and exclusion criteria are presented in Table 3. Studies were eligible for inclusion if they were published in English since 2012 and examined growth and HM intake outcomes of human milk‐fed preterm infants born <34 weeks gestational age or <1500 g who were discharged from the NICU with fortified HM feeds or supplemental PDF bottle feeds compared to unfortified HM or supplemental standard term formula bottle feeds. Eligible studies had at least 10 participants in each treatment group and were randomized controlled trials, retrospective chart reviews, or prospective or retrospective cohort or cross‐sectional study designs. Studies outside of these criteria were excluded. The 2012 cutoff for study inclusion was selected because our review builds upon the 2013 Cochrane review, whose literature search concluded in 2012. Prior to data extraction, this systematic review was registered with Prospero (CRD420251045513).^26^

**Table 3:** Full text inclusion and exclusion criteria.

	Inclusion	Exclusion
Population	*Target population*: Preterm infants born <34 weeks gestational age or <1500 g *Discharge feeding*: HM (expressed fresh or frozen mother's own milk, and/or directly BF) *Variable of interest*: Discharge feeding expressed HM fortified with multi‐nutrient HMF, expressed HM milk fortified with powder PDF, or directly BF with supplemental bottle feedings of PDF	*Target population*: Preterm infants born ≥34 weeks gestational age or ≥1500 g, late preterm infants, LBW infants > 2500 g, and term infants *Discharge feeding*: Enteral feeding via gastric tube, exclusive infant formula feeding, and no discharge feeding comparison group *Variable of interest*: Direct BF or expressed HM with no fortification, exclusive formula feeding at discharge
Sex	Male or female	
Health status	Premature birth (<34 weeks)	Premature birth >34 weeks gestational age or term birth
Outcome variables	*Aim 1*: Growth measurements in weight (g), length (cm), head circumference (cm), weight, length, and head circumference for age *z*‐scores, and change in growth *z*‐scores from discharge through 6 months and from discharge through 12 months CA *Aim 2*: HM intake outcomes as the proportion of study population consuming HM at the follow‐up period, proportion of feedings that are HM (direct BF or expressed HM), and duration of HM intake (postnatal or postdischarge weeks) from discharge through 6 months and from discharge through 12 months CA	No reported HM intake or growth outcomes after discharge
Study design	Randomized control trial, retrospective chart review, prospective or retrospective cohort, cross‐sectional	Case study
Publication year	Since 2012	Before 2012
Study group size	At least 10 in each group	<10 in each group
Country	Any	Any
Language	English	Any language other than English

Abbreviations: BF, breast feeding; CA, corrected age; CM, centimeter; g, gram; HM, human milk; HMF, human milk fortifier; LBW, low birth weight.

### Search strategy

This study adhered to the PRISMA guidelines for reporting (Tables S1 and S2.)^25^ A health sciences librarian (MG) collaborated with the research team to identify relevant keywords and develop the search strategy. The literature search inclusion criteria were restricted to articles in English, human studies, and publications from 2012 onward. The initial search was conducted in PubMed and Scopus, with keywords identified from titles, abstracts, and indexed terms using Medical Subject Headings (MeSH). A comprehensive search was then performed in CINAHL (EBSCO), Cochrane Library (Wiley), Dissertations & Theses Global (ProQuest), Embase (Elsevier), PubMed (NLM), Scopus (Elsevier), and Web of Science (Clarivate) (Table S4). Search results were assessed for relevance, with cited references and relevant reviews examined for additional studies. Publications by primary authors were also searched. Google Scholar was searched with the first 350 results retained. Reports and unpublished studies were sought from professional organizations (Table S4).

### Data collection

In total, 7500 records were identified through the database, grey literature, and hand citation searches and imported to EndNoteX20.^27^ After removing duplicates, 3146 citations remained and were shared by MG with one investigator (TR) via an EndNoteX20 library,^27^ which was then imported into Rayyan screening.^24^, ^28^ Two investigators (TR and AG) independently screened the titles and abstracts, resulting in 24 articles for full‐text review without the need of a third investigator to reach consensus. The 24 articles selected for full‐text screening were uploaded and independently screened in Rayyan^28^ by the same two investigators (TR and AG). Three articles required independent review from a third investigator (MN) to assist in achieving consensus. Twenty articles were excluded, and four articles were selected for inclusion in this systematic review (Figure 2 **)**.^29^, ^30^, ^31^, ^32^

**Figure 2:**
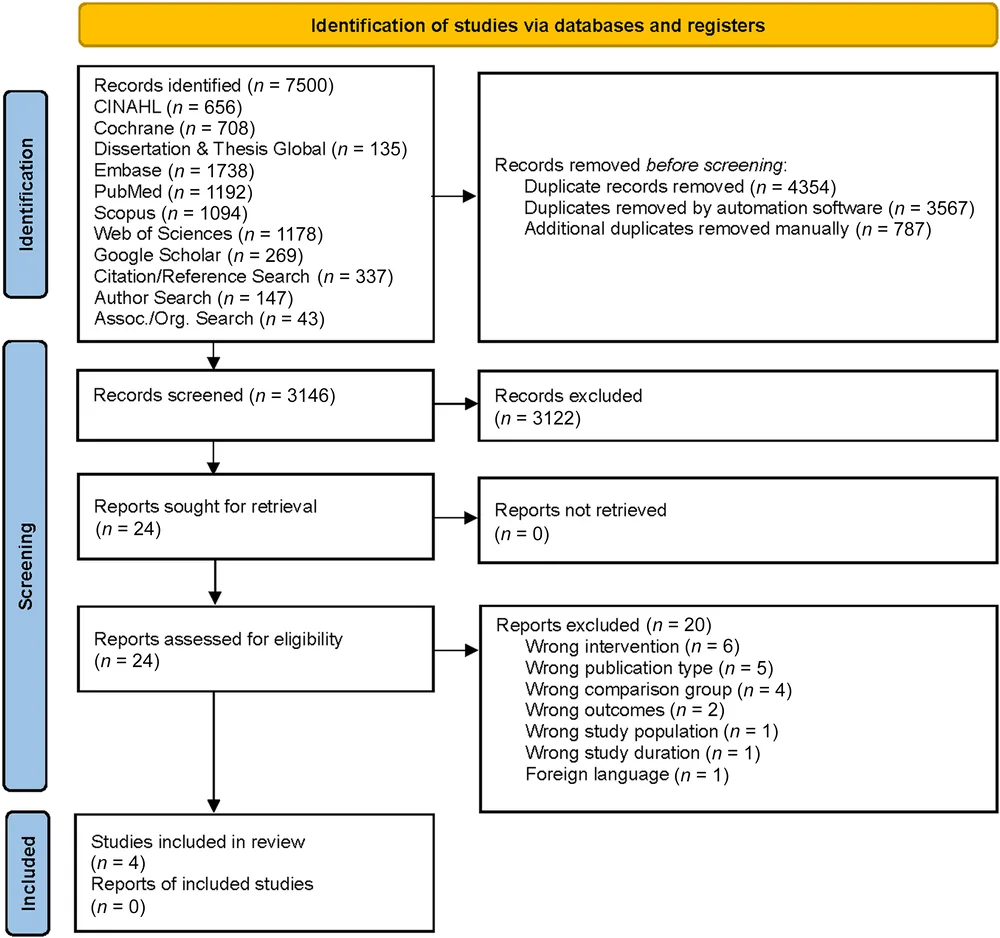
PRISMA 2020^25^ flow diagram of literature search, full‐text screening and study selection process.

### Data extraction

Data from each study was extracted by one investigator (TR) and was reviewed for correctness by a co‐investigator (AG). An Excel workbook was developed, modeled after the data collection form from the *Cochrane Handbook for Systematic Reviews of Interventional Studies*.^24^ Data extracted included the study characteristics (citation, country, study design, sample size, and funding source), participant characteristics (gestational age at birth, birth anthropometrics—weight, length, HC, *z*‐scores, gestational age at follow‐up), feeding method at discharge, and data relating to outcomes of interest: (1) growth measurements weight (g), length (cm), HC (cm), weight, length and HC‐for‐age *z*‐scores and change in growth *z*‐scores from discharge through 6 months CA and from discharge through 12 months CA; and (2) HM intake outcomes of the proportion of study population consuming any HM, proportion of daily feeding volume as HM, and the duration of HM intake in postdischarge weeks.

### Risk‐of‐bias assessment

Two investigators (TR and AG) independently assessed the risk of bias in each study using the risk‐of‐bias tool for randomized trials version 2 (RoB 2)^24^ for the two randomized controlled trials (RCTs) and the Risk of Bias in Non‐randomized Studies of Interventions version 2 (ROBINS‐I v2)^33^ for the two non‐randomized control studies. These tools were selected to ensure methodological consistency as the current domain‐based frameworks recommended by the *Cochrane Handbook* for a rigorous, standardized assessment of internal validity across all study designs.^24^ The RoB 2 evaluated the risk of bias in the randomization process, intervention deviations, missing outcomes data, outcomes measurements, and selection of the reported results, assessing the risk of bias as low, some concerns, or high.^24^ The ROBINS‐I v2 evaluated the risk of bias due to confounding, classification of interventions, in the selection of participants for inclusion in the study or analysis, intervention deviations, missing data, outcomes measurements, and selection of the reported results, assessing the risk of bias as low, moderate, serious, and critical.^33^ The risk of bias assessments were reviewed and agreed upon by the co‐investigators.

### Data synthesis and assessment of heterogeneity

Although all studies reported one or more growth or HM intake outcomes, the method of reporting and timing of measurement varied between studies. Since there was no overlap in the reported measurements of growth and HM intake outcomes or timing of measurement, a meta‐analysis was not possible. Additionally, due to the heterogeneity between studies in study design, feeding interventions, and reporting of outcomes, a formal assessment of the certainty of evidence for each outcome was not performed for this systematic review. Study findings were synthesized narratively and are presented in Table 4.

**Table 4:** Overview of study characteristics.

				Discharge diet		
Author, Year, Country	Design	GA & BW inclusion (*n*)	GA (wk) and BW (g)^a^	Intervention	Comparison	Outcomes measurements of interest
Lok et al., 2024, USA^30^	Retrospective cohort	≤30 wk GA (*n* = 296)	I: 27.6 (±1.9), 1090.0 (±317.8) C: 27.9 (±2.0) 1151.0 (±314.3)	FHM or BF+PDF^b^ *n* = 183	Unfortified HM *n* = 113	Growth: wt‐for‐age *z*‐score, wt‐for‐age *z*‐score change since d/c at first f/u^c^ HM intake: proportion of study group consuming HM at first f/u^c^
Filho et al., 2021, Brazil^32^	Parallel unblinded RCT	<1500 g and <37 wk GA, BF at discharge (*n* = 58)	I: 31.1 (±2.3), 1281.0 (±196.3) C: 30.7 (±2.4), 1312.3 (±171.6)	FHM^d^ *n* = 30	Unfortified HM *n* = 28	Growth: change in wt‐, ln‐, and HC‐for‐age *z*‐score at 6 mo CA^e^ HM intake: proportion of study group consuming HM at 6 mo CA
Toftlund et al., 2018, Denmark^31^	Prospective, unblinded RCT 6‐year follow‐up	<33 wk GA (*n* = 150)	I: 29.6 (24.1–32.0), 1328.0 (535.0–2014.0) C: 30.3 (24.4–32.0), 1260.0 (548.0–2255.0)	FHM^f^ *n* = 79	Unfortified HM *n* = 71	Growth: wt‐ and ln‐for‐age *z*‐score and, change in wt‐ and ln‐for‐age *z*‐score at 6 and 12 mo CA^g^ HM intake: not assessed
Japakadetr et al., 2016, Thailand^29^	Prospective, non‐randomized interventional cohort	<34 wk and BW 750–1499 g (*n* = 44)	I: 29.0 (±1.7), 1192.0 (±202.0) C: 30.0 (±2.0), 1151.0 (±208.0)	FHM or BF+PDF^h^ *n* = 22	Unfortified HM or BF + STF *n* = 22^i^	Growth: achieved wt (g), HC (cm) and wt‐, ln, and HC‐for‐age *z*‐score at 2 and 6 mo CA HM intake: proportion of study group consuming HM and duration (wks since d/c) at 2 and 6 mo CA

Abbreviations: BC, body composition; BF, breastfeeding; C, comparison; d/c, discharge; EN, enteral nutrition; FHM, fortified human milk; FM, fat mass; f/u, follow‐up; GA, gestational age; HC, head circumference; I, intervention; ln, length; mo, months; PMA, postmenstrual age; RCT, randomized control trial; STF, standard term formula; wk, weeks; wt, weight, wt.

^a^ Mean (±SD) or median (range).

^b^ Mixed diet described as human milk with formula and/or added calories (products not described); 84.2% of participants consumed human milk fortified or supplemented to ≥22 kcal/oz.

^c^ First follow‐up occurred between 1 and 4 months after discharge; mean (SD) corrected age (months) at follow‐up: intervention group 1.3 (±0.9), comparison group 1.2 (±0.9).

^d^ Fortified with PreNan (Nestlé, Vevey, Switzerland) powder preterm postdischarge formula 2 g in 10 mL human milk twice per day (+20 kcal and 0.6 g protein/day).

^e^ Change in monthly anthropometric growth for age *z*‐scores in unadjusted longitudinal mixed‐effects model; outcomes only reported for 6 months corrected age.

^f^ Fortified with powder multi‐nutrient human milk fortifier, five packs to 20–50 mL human milk in one feeding (17.5 kcal, 1.4 g protein).

^g^ Outcomes were reported at 2, 4, 6, and 12 months CA; present study reporting outcomes at 6 and 12 months corrected age.

^h^ Human milk fortified with powder PreNan preterm postdischarge formula to 22 kcal/oz (*n* = 18/81.1%) and/or mixed feeding of human milk and postdischarge formula (22 kcal/oz).

^i^ Historical controls.

### Quality assessment and quality control

The development of this study was guided by the *Cochrane Handbook on Systematic Reviews for Interventions*.^24^, ^34^ A health sciences librarian (MG), as part of the research team, developed the search strategy and conducted a comprehensive literature search, including grey literature and hand citation search. The research team met regularly to align on the progress of the research project, the methodology, and to address and resolve any questions or issues that were raised during the process. Additionally, all studies were critically appraised for quality and risk of bias.

## RESULTS

### Literature search and study characteristics

Four studies met eligibility criteria: two randomized controlled trials^31^, ^32^ and two cohort studies.^29^, ^30^ An overview of the study characteristics is presented in Table 4. Results were reported on a total of 548 participants discharged with fortified HM (*n* = 109),^31^, ^32^ fortified HM or direct BF with PDF bottle feeds (*n* = 205),^29^, ^30^ unfortified HM (*n* = 212),^30^, ^31^, ^32^ and unfortified HM or direct BF with standard term formula bottle feeds (*n* = 22).^29^ The study designs and feeding interventions varied; one study was a retrospective cohort study,^30^ one was a parallel, unblinded RCT,^32^ one was a 6‐year follow‐up of a previously published, prospective, unblinded RCT,^31^ and one was a prospective, non‐randomized interventional cohort study.^29^ Discharge feeding interventions varied as well: three studies fortified HM with PDF^29^, ^30^, ^32^ while one used a multi‐component HMF.^31^ Three studies compared fortified HM with unfortified HM feedings,^30^, ^31^, ^32^ and one study's comparison group included mixed feeding of BF and standard term formula; however, the majority (77.3%) were exclusively fed HM.^29^ Across the four studies,^29^, ^30^, ^31^, ^32^ assessments of growth and HM intake outcomes varied in both methods of reporting data and timing of outcome measurements, ranging from 1 to 12 months CA.

### Growth outcomes

#### Weight

Weight growth outcomes are presented in Table 5. Among the included studies, only Japakasetr et al.^29^ reported that, although the achieved weight was greater in the fortified group (4358.0 ± 835.0 g), it did not differ significantly from the unfortified group (3892.0 ± 797.0 g, *p* > 0.05) at 2 months CA. At 6 months CA the fortified group had a significantly greater achieved weight than the unfortified group (6889.0 ± 869.0 g vs 5910.0 ± 996.0 g, *p* < 0.001).^29^ Japakasetr et al.^29^ also found that 90.9% (*n* = 20) of the fortified group had weight *z*‐score > −2SD at 6 months CA compared to 50.0% (*n* = 11) of the unfortified group (*p* = 0.007).

**Table 5:** Weight, length, and head circumference growth outcomes.^a^

	**Lok et al., 2024** ^30^ ^,^ b I: *n* = 183 C: *n* = 113	**Filho et al., 2021** ^32^ ^,^ c I: *n* = 30 C: *n* = 28	**Toftlund et al., 2018** ^31^ ^,^ d I: *n* = 79 C: *n* = 71	**Japakasetr et al., 2016** ^29^ ^,^ e I: *n* = 22 C: *n* = 22
**Weight**				
Weight (g) mean (SD)				
1st f/u				I: 4358 (±835) C: 3892 (±797) ns
2nd f/u				I: 6889 (±869) C: 5910 (±996) *p* < 0.001
Weight‐for‐age	Percentile mean (SD):		*z*‐score trend mean:	*z*‐score > −2SD *n* (%)
1st f/u	I: 46.4 (±32.9) C: 38.9 (±31.1) ns		I: ↑ C: ↓ ns	I: 14 (63.6%) C: 9 (40.9%) ns
2nd f/u			I: ↑ C: ↓ ns	I: 20 (90.9%) C: 11 (50.0%) *p* = 0.007
Δ Weight‐for‐age	Percentile mean (SD):	Association of intervention to outcome^c^:	*z*‐score trend mean:	
Discharge to 1st f/u	I: 9.9 (±27.8) C: 7.3 (±23.6) ns	0.234 [95% CI: 0.099, 0.368] *P* = 0.001	I: ↑ C: ↓ ns	
Discharge to 2nd f/u			I: ↑ C: ↓ ns	
**Length**				
Length‐for‐age			*z*‐score trend mean:	*z*‐score > −2SD *n* (%)
1st f/u			I: ↓ C: ↑ ns	I: 15 (68.2%) C: 8 (36.4%) ns
2nd f/u			I: ↓ C: ↑ ns	I: 17 (77.3%) C: 11 (50.0%) ns
Δ Length‐for‐age		Association of intervention to outcome^b^:	*z*‐score trend mean:	
Discharge to 1st f/u		0.058 [95% CI: −0.157, 0.272] ns	I: ↓ C: ↑ ns	
Discharge to 2nd f/u			I: ↓ C: ↑ ns	
**Head circumference**				
HC (cm) mean (SD)				
1st f/u				I: 37.3 (±1.5) C: 36.7 (±2.3) ns
2nd f/u				I: 43.5 (±2.0) C: 40.8 (±2.1) *p* < 0.001
HC‐for‐age				*z*‐score > −2SD *n* (%)
1st f/u				I: 17 (77.3%) C: 10 (45.5%) ns
2nd f/u				I: 21 (95.5%) C: 12 (54.5%) *p* = 0.004
Δ HC‐for‐age		Association of intervention to outcome^b^:		
Discharge to 1st f/u		−0.657 [95% CI: −0.168, 0.373] ns		

Abbreviations: C, comparison group; f/u, follow‐up; I, intervention group; ns, not significant; SD, standard deviation.

^a^ Values reported as *n* (%), mean (±SD), or median (IQR); not significant (ns) is *p* > 0.05.

^b^ First follow‐up visit occurred 1–4 months after discharge with mean (SD) corrected age (months): intervention 1.3 (±0.9), comparison 1.2 (±0.9).

^c^ Outcome reported as mixed‐effects linear model for association between intervention and outcome from discharge to 6 months corrected age.

^d^ Presenting the 6 months corrected data for first follow‐up and 12‐months corrected age data for second follow‐up; study outcomes were reported graphically, raw data not available, and arrows represent the trend line direction.

^e^ First and second follow‐up visits at 2 months and 6 months corrected age, respectively; *z*‐score‐for‐age reported as *n* (%) *z*‐score > −2SD.

Across the included studies, reporting of *z*‐scores was notably limited; only Toftlund et al.^31^ reported weight‐for‐age *z*‐scores and change in *z*‐score outcome; however, the data were only presented in graphical form, finding no significant differences in either metric between the fortified HM and unfortified HM feeding groups at 6 and 12 months CA. Lok et al.^30^ evaluated weight outcomes using percentiles rather than z‐scores or raw anthropometric values, finding at the first follow‐up visit (mean 1.2–1.3 months CA) that the fortified group had a higher mean weight percentile (46.4 ± 32.9) compared to the unfortified group (38.9 ± 31.1), although this difference did not reach statistical significance (*p* = 0.116). Similarly, the change in weight percentile from NICU discharge to the first follow‐up visit was greater in the fortified group (+9.9 ±  27.8) versus the unfortified group (+7.3 ± 23.6), again without statistical significance (*p* = 0.217).^30^ Filho et al.^32^ found a significant association between the fortified feedings and change in weight‐for‐age *z*‐score (0.234 [95% CI: 0.099, 0.368], *p* = 0.001) at 6 months CA, meaning that fortified feedings had a greater impact on the change in weight from discharge to 6 months CA than unfortified feedings.

#### Length

Among the three studies reporting length, none found significant differences between groups in length growth outcomes (Table 5).^29^, ^31^, ^32^ However, among the fortified group in Japakasetr et al.^29^ a greater percentage of infants achieved a length *z*‐score greater than −2SD compared to the unfortified group.

#### Head circumference

Growth outcomes for HC are presented in Table 5, with Japakasetr et al.^29^ reporting achieved HC (cm) at follow‐up, finding that the fortified group had significantly higher achieved HC than the unfortified group (43.5 ± 2.0 cm vs 40.8 ± 2.1 cm, *p* < 0.001), representing a mean difference of 2.7 cm at 6 months CA.^29^ The investigators also found that the proportion of participants with HC greater than −2SD was higher in the fortified group at 2 and 6 months CA, finding a significant difference at the latter follow‐up point (95.5% vs 54.4%, *p* = 0.004).^29^ Filho et al.^32^ did not find a significant association of fortified feedings and change in HC‐for‐age *z*‐score at six months CA.

Reporting of energy and protein intake across studies was variable and did not consistently permit calculation of total achieved intake differences between groups.^29^, ^30^, ^31^, ^32^ Toftlund et al.^31^ and Filho et al.^32^ reported only the incremental contribution of fortification (+17.5 kcal and 1.4 g protein/day; +20 kcal and 0.6 g protein/day, respectively), while Lok et al.^30^ described feeds fortified to ≥22 kcal/oz without quantifying total intake. Only Japakasetr et al.^29^ reported achieved intake per kilogram, demonstrating significantly higher median energy and protein intake in the fortified group at 2, 4, and 6‐months CA (approximately 2.8–12.9 kcal/kg/day and 0.3–0.7 g protein/kg/day greater than controls; all *p* < 0.05). Thus, while precise pooled intake differences cannot be estimated, fortified strategies consistently provided modest increases in energy and protein relative to unfortified feeding.

### Human milk intake outcomes

Three of the four studies reported HM intake outcomes (Table 6).^29^, ^30^, ^32^ Lok et al.^30^ and Filho et al.^32^ measured the proportion of infants receiving any HM at the respective study follow‐up points. In contrast, Japakasetr et al.^29^ reported the proportion of daily feeds as HM at 2 and 6 months CA, and the duration of HM intake in weeks since discharge.

**Table 6:** Human milk intake outcomes.

	**Loket al., 2024** ^30^ ^,^ ^b^ I: *n* = 183 C: *n* = 113	**Filho et al., 2021** ^32^ ^,^ ^c^ I: *n* = 30 C: *n* = 28	**Toftlund et al., 2018** ^31^ ^,^ ^d^ I: *n* = 79 C: *n* = 71	**Japakasetr 2016** ^29^ ^,^ ^e^ I: *n* = 22 C: *n* = 22
Participants consuming HM *n* (%)				
1st f/u	I: 135 (73.8%) C:83 (73.5%) ns	I: 24 (80.9%) C: 22 (78.2%) ns	n/a	
Proportion of daily feeds as HM median (IQR)				
1st f/u			n/a	I: 35.5% (10.2, 79.2) C: 48.5% (20.2, 120.21) *ns*
2nd f/u				I: 11.8% (4.4, 16.4) C: 2.3% (0.6, 7.9) *p* = 0.041
Duration of HM intake median (IQR)				
Weeks since discharge			n/a	I: 16.0 (10.0, 26.0) C: 18.0 (12.0, 24.0) ns

Abbreviations: C, comparison group; f/u, follow‐up; IQR, interquartile range; HM, human milk; I, intervention group; ns, not significant.

^a^ Not significant (ns) is *p* > 0.05.

^b^ First follow‐up visit occurred 1–4 months after discharge with mean (SD) corrected age (months): intervention group 1.3 (±0.9), comparison group 1.2 (±0.9).

^c^ Outcome reported at 6 months corrected age.

^d^ Human milk intake outcomes not reported.

^e^ First and second follow‐up visits at 2 months and 6 months corrected age, respectively.

#### Proportion of population consuming HM

Lok et al.^30^ reported that the majority of participants in both groups were consuming some HM intake at the first follow‐up (mean 1.2–1.3 months CA), finding similar rates of HM consumption between the fortified group (73.8%) and the unfortified group (73.5%). Filho et al.^32^ reported similar rates of HM intake, finding that the majority of both groups (80.9% vs 78.2%) were consuming some HM intake at 6 months CA.

#### HM feeding daily volume and duration

Only Japakasetr et al.^29^ quantified HM intake as a percentage of daily feeds at follow‐up, finding no differences at 2 months CA (35.5% vs 48.5%). Although the proportion of daily feeds declined at 6 months CA, it was significantly higher in the fortified group at a median (interquartile range) 11.8% (4.4–16.4) versus 2.3% (0.6–7.9, *p* = 0.041) in the unfortified group.^29^ Duration of HM intake was reported as weeks of exposure only in Japakasetr et al.^29^ Although the fortified group's median HM intake duration was 2 weeks shorter than the unfortified group, the difference was not statistically significant.^29^

### Risk of bias and quality assessment

Study quality and risk of bias were assessed using RoB 2 for the two intervention trials, and ROBINS‐I v2 for the two observational studies. Three studies received scores of low risk^29^, ^30^, ^31^ and one study received a score of some risk due to lack of blinding; results were only reported on the participants who returned for follow‐up assessment, and intent‐to‐treat analysis was not conducted^32^ (Figures 3 and 4).

**Figure 3:**
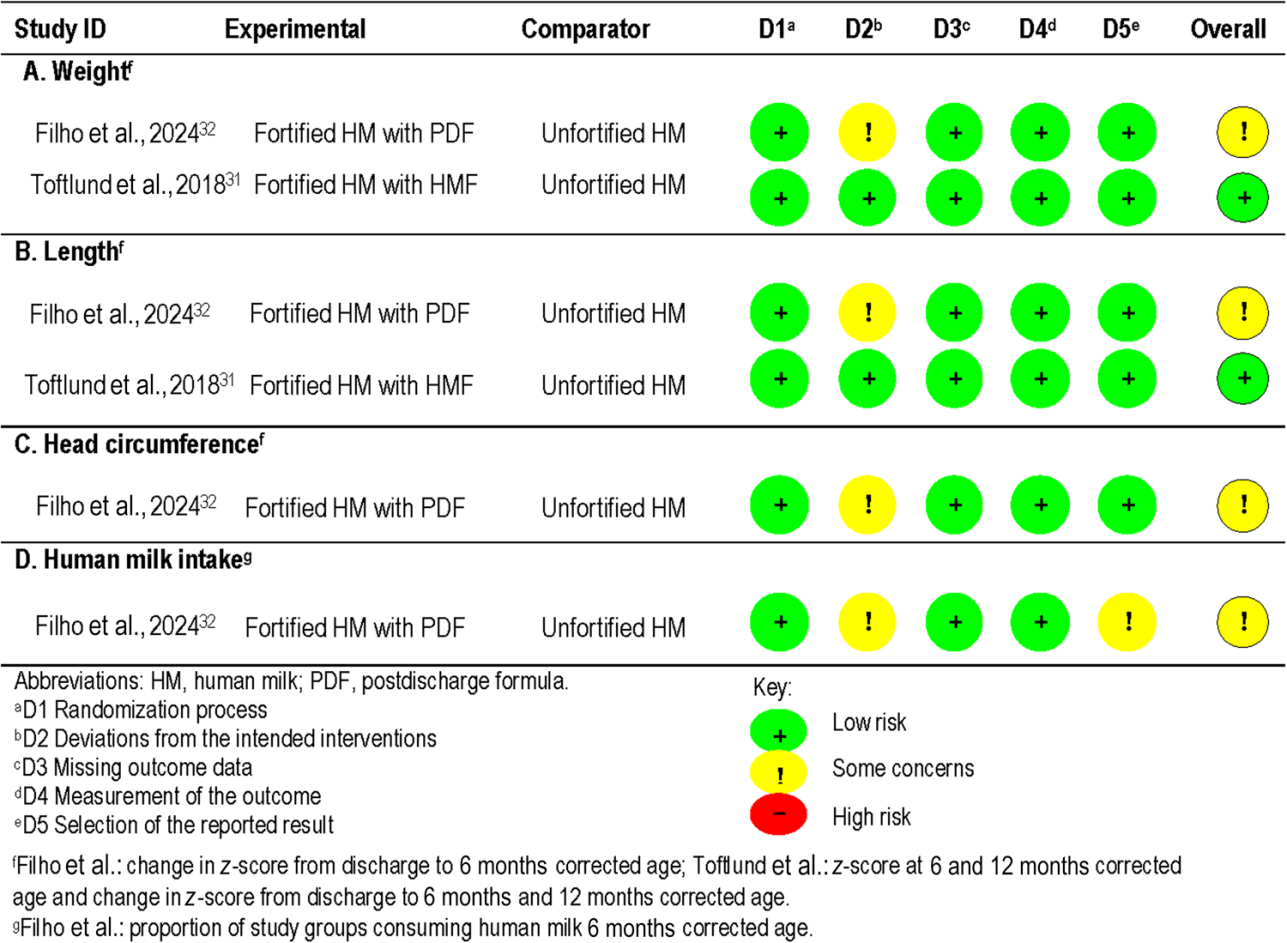
Risk‐of‐bias 2 (RoB 2) assessment results by outcome.

**Figure 4:**
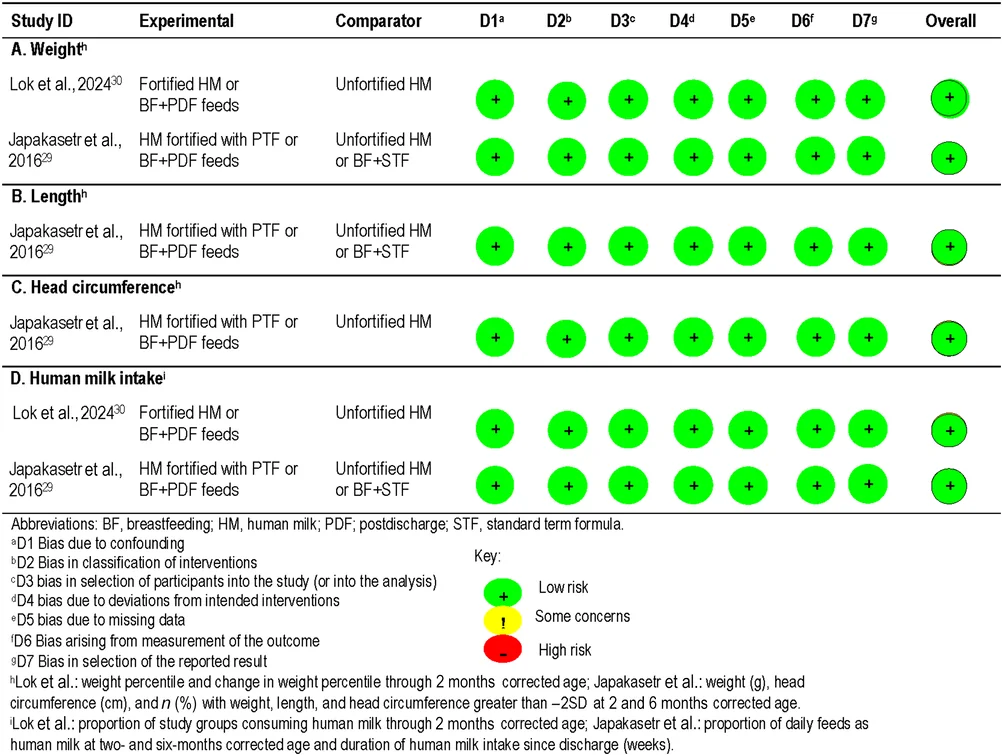
Risk‐of‐bias in non‐randomized studies (ROBINS‐I v2) assessment results by outcome.

## DISCUSSION

This systematic review examined the effects of postdischarge fortified expressed HM feedings and/or direct BF with PDF bottle feeds compared to unfortified HM or direct BF with standard term formula bottle feeds on growth and HM intake outcomes in preterm infants born <34 weeks gestation or <1500 g. Four studies met eligibility criteria (two RCTs, two cohort studies).^29^, ^30^, ^31^, ^32^

### Growth

Reporting was varied across studies, limiting comparability; however, all four demonstrated improved weight with postdischarge HM fortification or BF plus PDF bottle feeds, with two showing significant gains at 6 months CA.^29^, ^30^, ^31^, ^32^ One study also reported significantly higher achieved HC (approximately 2.7 cm) and greater proportion of infants with HC above −2SD.^29^ Prior studies of fortified HM and early provision of high protein intake have demonstrated improved HC growth; however, this evidence reflects in‐hospital nutrition rather than postdischarge interventions.^1^, ^9^ The observed difference in achieved HC at 6 months CA should therefore be interpreted cautiously, as it was reported in a single study with a modest sample size.^29^ Replication in larger, well‐designed prospective studies is needed to determine whether similar HC differences are consistently observed with postdischarge fortification.

While growth trajectories were similar between groups at 2 months CA,^29^, ^30^ significant differences in weight and HC emerged by 6 months CA in the fortified groups,^29^, ^31^, ^32^ suggesting that longer duration of postdischarge nutrient enrichment may be necessary to observe measurable effects. In contrast to the 2013 Cochrane review, which found no growth benefit from 3 to 4 months of postdischarge HMF fortification,^21^ our review reflects current postdischarge feeding practices and demonstrated improvements across multiple growth measures at 6 months CA.^29^, ^30^, ^32^

The nutrient advantage of fortified HM or BF with PDF bottle feeds (22–24 kcal/oz; 10%–20% more energy, 25%–86.7% more protein than unfortified HM or standard formula)^8^, ^20^ supports the expectation of greater growth. In the study reporting median nutrition intake, fortified feeds provided 18.6% more energy and 20.9% more protein per kilogram than unfortified feeding at 6 months CA,^29^ which may partially explain the observed growth differences. However, variability in reported nutrition intake across studies limits our ability to determine how much these intake differences contributed to growth outcomes.

### Human milk intake

Across the three studies reporting HM intake, postdischarge HM fortification or BF with PDF bottle feeds generally maintained HM intake,^29^, ^30^, ^32^ with some evidence of greater HM intake in the fortified groups.^29^ High proportions of infants continued to consume HM at follow‐up regardless of feeding strategy,^30^, ^32^ and Japakasetr et al.^29^ found a significantly greater proportion of daily feeds as HM with fortification or supplemental PDF feeds. Postdischarge fortification and PDF supplementation are intended to meet preterm infants’ increased nutritional needs without displacing HM,^8^, ^9^ yet parental or caregiver perceptions of infant formula as superior may lead to it replacing HM.^21^ While Ericson et al.^22^ reported that partial exclusive BF at NICU discharge was associated with reduced or ceased BF by 2–6 months postnatal age, the effect of HM fortification or PDF supplementation was not evaluated.^22^ This systematic review builds upon prior literature by demonstrating that fortified feeding strategies generally preserved HM intake, with outcomes comparable to unfortified through 6 months CA.^29^, ^30^, ^32^

### Barriers and limitations

This review has several limitations. First, only four studies met the inclusion criteria,^29^, ^30^, ^31^, ^32^ and one study exhibited some risk of bias due to lack of blinding and incomplete outcome reporting.^32^ Second, the heterogeneity in feeding interventions, outcome measures, and follow‐up timing precluded conducting a meta‐analysis and limited direct comparisons. Products used for fortification varied among studies: one used commercial HMF,^31^ two used PDF for fortification,^29^, ^32^ and one did not specify the product.^30^ The interventions also varied in whether infants received fortified expressed HM only,^31^, ^32^ or a combination of fortified HM and direct BF with supplemental PDF feeds.^29^, ^30^ Although each study used standard procedures to obtain weight, length, and HC measurements, the method of analyzing and reporting outcomes varied between groups, limiting direct comparisons.^29^, ^30^, ^31^, ^32^ Another limitation is the lack of consistent reporting on *z*‐scores and other standardized growth metrics. These inconsistencies limit the ability to interpret growth trajectories and generalize findings across populations.^29^, ^30^, ^31^, ^32^ Additionally, HM intake outcomes were not uniformly defined or measured.^29^, ^30^, ^31^, ^32^ Only one study reported HM intake as a proportion of daily feeds or in weeks of duration.^29^ These metrics are critical for evaluating the impact of postdischarge strategies on sustained HM exposure. Generalizing outcomes from this systematic review is limited to stable preterm infants, since the studies excluded very sick or high‐risk preterm infants who may have comorbidities at discharge that impact their dietary intake and growth independent of their discharge feeding protocol.^3^, ^8^ Although a comprehensive search strategy was developed by the research team, which included a health sciences librarian, relevant studies may have been missed, potentially limiting this study's findings. Finally, outcomes beyond growth and HM intake outcomes were not evaluated in this systematic review.

### Clinical implications

Expert consensus guidelines from the ESPGHN,^8^ the AAP,^17^ the AND,^15^ and the European Milk Bank Association^9^ emphasize an HM‐based diet and recommend postdischarge fortification of expressed HM or PDF feeding for preterm infants with altered nutrition status or postnatal growth failure. The findings of this review have important implications for nutrition practices for preterm infants after NICU discharge. This is the first systematic review, to our knowledge, to include studies using HM fortified with PDF and direct BF with supplemental PDF bottle feeds. The results support the continued postdischarge feeding of HM using fortification strategies to promote catch‐up growth in preterm infants. Clinicians should consider these feeding options, particularly for infants with malnutrition or postnatal growth failure at NICU discharge. This review underscores the need for standardized guidelines on postdischarge HM fortification practices to reduce the wide variability in feeding practices observed in clinical settings^3^, ^6^, ^9^, ^15^, ^16^, ^17^, ^19^ and support the expert consensus recommendation of continued nutritional support.^3^, ^8^, ^15^, ^17^

Future research should use rigorously designed prospective studies comparing specific postdischarge HM fortification strategies and measurement of HM intake. The use of both short‐ and long‐term follow‐up assessment of standardized outcome measures (weight, length, HC, growth *z*‐scores, *z*‐score changes, and growth velocity) and clear descriptions of feeding interventions—including product type, nutrient composition, and separate reporting for mixed strategies—will improve comparability and support meta‐analyses. Given the wide variation in intervention duration (1.2–12 months CA), further work should determine the optimal length of postdischarge fortified feeding. Additionally, outcomes beyond anthropometric growth, such as bone mineralization and bone density, should be explored. While randomization may not always be feasible, unfortified HM‐fed comparison groups are essential, and outcomes should be assessed both early (≤6 months CA) and longer term (to 12 months CA or beyond) to define the enrichment period needed to support catch‐up growth. These efforts when designing future studies will improve comparability and support future meta‐analyses.

## CONCLUSION

This systematic review suggests that postdischarge HM fortification strategies may enhance growth in preterm infants, particularly for weight and HC at 6 months CA, without reducing HM intake. Fortified feeding approaches may therefore support the higher nutritional needs of preterm infants after discharge, and PDF may serve as a practical fortifier when commercial HMFs are unavailable. However, the evidence remains limited and heterogeneous due to methodological variability and the small number of eligible studies. As discharge increasingly emphasizes HM‐based diets, the variability in our findings underscores the need for rigorous studies to further evaluate the impact of postdischarge HM fortification and supplemental PDF feeds on growth, particularly linear growth, as well as HM intake outcomes.

## AUTHOR CONTRIBUTIONS

Tara Rebele, Allison Gomes, Melanie Newkirk, Laura Byham‐Gray, and Jane Ziegler equally contributed to the conception and design of the research; Tara Rebele, Allison Gomes, Melanie Newkirk, and Mina Ghajar contributed to the acquisition and analysis of the data; Tara Rebele, Allison Gomes, Melanie Newkirk, Laura Byham‐Gray, and Jane Ziegler contributed to the interpretation of the data; and Tara Rebele drafted the manuscript. All authors critically revised the manuscript, agreed to be fully accountable for ensuring the integrity and accuracy of the work, and read and approved the final manuscript.

## CONFLICT OF INTEREST STATEMENT

Tara Rebele is employed by BASF Corporation and serves on the Mead Johnson Nutrition Speaker's Bureau. The remaining authors declare no conflicts of interest.

## Supporting information

Supplementary Material Postdischarge Nutrition SR NCP.
