# Supplementary material for: Growth and human milk intake outcomes from postdischarge feedings among very‐low‐birth‐weight preterm infants: A systematic review
Source: Nutr Clin Pract. 2026 Apr 29;41(5):1334–48. doi: 10.1002/ncp.70127 (PMC13622408; doi:10.1002/ncp.70127)
Supplement: Supplementary file 1 [file NCP-41-1334-s001.pdf]

Table S1: PRISMA 2020 checklist for systematic review[24]

Section and Topic	Item #	Checklist item	Location where item is reported
**TITLE**			
Title	1	Identify the report as a systematic review.	Title page
**ABSTRACT**			
Abstract	2	See the PRISMA 2020 for Abstracts checklist.	Page 1
**INTRODUCTION**			
Rationale	3	Describe the rationale for the review in the context of existing knowledge.	Page 3
Objectives	4	Provide an explicit statement of the objective(s) or question(s) the review addresses.	Page 4
**METHODS**			
Eligibility criteria	5	Specify the inclusion and exclusion criteria for the review and how studies were grouped for the syntheses.	Page 4, Table 3
Information sources	6	Specify all databases, registers, websites, organisations, reference lists and other sources searched or consulted to identify studies. Specify the date when each source was last searched or consulted.	Page 5, Table S4
Search strategy	7	Present the full search strategies for all databases, registers and websites, including any filters and limits used.	Table S4
Selection process	8	Specify the methods used to decide whether a study met the inclusion criteria of the review, including how many reviewers screened each record and each report retrieved, whether they worked independently, and if applicable, details of automation tools used in the process.	Pages 5-6, Figure 2
Data collection process	9	Specify the methods used to collect data from reports, including how many reviewers collected data from each report, whether they worked independently, any processes for obtaining or confirming data from study investigators, and if applicable, details of automation tools used in the process.	Pages 5-6
Data items	10a	List and define all outcomes for which data were sought. Specify whether all results that were compatible with each outcome domain in each study were sought (e.g. for all measures, time points, analyses), and if not, the methods used to decide which results to collect.	Pages 5-6
	10b	List and define all other variables for which data were sought (e.g. participant and intervention characteristics, funding sources). Describe any assumptions made about any missing or unclear information.	Pages 5-6, Table S3
Study risk of bias assessment	11	Specify the methods used to assess risk of bias in the included studies, including details of the tool(s) used, how many reviewers assessed each study and whether they worked independently, and if applicable, details of automation tools used in the process.	Page 6
Effect measures	12	Specify for each outcome the effect measure(s) (e.g. risk ratio, mean difference) used in the synthesis or presentation of results.	n/a
Synthesis methods	13a	Describe the processes used to decide which studies were eligible for each synthesis (e.g. tabulating the study intervention characteristics and comparing against the planned groups for each synthesis (item #5)).	Pages 5-7, Table 3, Table S3

Section and Topic	Item #	Checklist item	Location where item is reported
	13b	Describe any methods required to prepare the data for presentation or synthesis, such as handling of missing summary statistics, or data conversions.	n/a
	13c	Describe any methods used to tabulate or visually display results of individual studies and syntheses.	Tables 4 & 5
	13d	Describe any methods used to synthesize results and provide a rationale for the choice(s). If meta-analysis was performed, describe the model(s), method(s) to identify the presence and extent of statistical heterogeneity, and software package(s) used.	Page 7
	13e	Describe any methods used to explore possible causes of heterogeneity among study results (e.g. subgroup analysis, meta-regression).	n/a
	13f	Describe any sensitivity analyses conducted to assess robustness of the synthesized results.	n/a
Reporting bias assessment	14	Describe any methods used to assess risk of bias due to missing results in a synthesis (arising from reporting biases).	n/a
Certainty assessment	15	Describe any methods used to assess certainty (or confidence) in the body of evidence for an outcome.	n/a
**RESULTS**			
Study selection	16a	Describe the results of the search and selection process, from the number of records identified in the search to the number of studies included in the review, ideally using a flow diagram.	Page 5, Figure 2
	16b	Cite studies that might appear to meet the inclusion criteria, but which were excluded, and explain why they were excluded.	Pages 5-6, Figure 2
Study characteristics	17	Cite each included study and present its characteristics.	Pages 6-7, Table 4
Risk of bias in studies	18	Present assessments of risk of bias for each included study.	Page 11, Figures 3-4
Results of individual studies	19	For all outcomes, present, for each study: (a) summary statistics for each group (where appropriate) and (b) an effect estimate and its precision (e.g. confidence/credible interval), ideally using structured tables or plots.	Tables 5
Results of syntheses	20a	For each synthesis, briefly summarise the characteristics and risk of bias among contributing studies.	Pages 7-11, Figures 3-4
	20b	Present results of all statistical syntheses conducted. If meta-analysis was done, present for each the summary estimate and its precision (e.g. confidence/credible interval) and measures of statistical heterogeneity. If comparing groups, describe the direction of the effect.	n/a
	20c	Present results of all investigations of possible causes of heterogeneity among study results.	n/a

Section and Topic	Item #	Checklist item	Location where item is reported
	20d	Present results of all sensitivity analyses conducted to assess the robustness of the synthesized results.	n/a
Reporting biases	21	Present assessments of risk of bias due to missing results (arising from reporting biases) for each synthesis assessed.	n/a
Certainty of evidence	22	Present assessments of certainty (or confidence) in the body of evidence for each outcome assessed.	n/a
**DISCUSSION**			
Discussion	23a	Provide a general interpretation of the results in the context of other evidence.	Pages 11-14
	23b	Discuss any limitations of the evidence included in the review.	Pages 12-13
	23c	Discuss any limitations of the review processes used.	Pages 12-13
	23d	Discuss implications of the results for practice, policy, and future research.	Pages 13-14
**OTHER INFORMATION**			
Registration and protocol	24a	Provide registration information for the review, including register name and registration number, or state that the review was not registered.	Pages 5
	24b	Indicate where the review protocol can be accessed, or state that a protocol was not prepared.	Pages 5
	24c	Describe and explain any amendments to information provided at registration or in the protocol.	n/a
Support	25	Describe sources of financial or non-financial support for the review, and the role of the funders or sponsors in the review.	Title page
Competing interests	26	Declare any competing interests of review authors.	Title page
Availability of data, code and other materials	27	Report which of the following are publicly available and where they can be found: template data collection forms; data extracted from included studies; data used for all analyses; analytic code; any other materials used in the review.	n/a

Table S2: PRISMA 2020 abstract checklist for systematic review[24]

Section and Topic	Item #	Checklist item	Reported (Yes/No)
**TITLE**			
Title	1	Identify the report as a systematic review.	Yes
**BACKGROUND**			
Objectives	2	Provide an explicit statement of the main objective(s) or question(s) the review addresses.	Yes
**METHODS**			
Eligibility criteria	3	Specify the inclusion and exclusion criteria for the review.	Yes
Information sources	4	Specify the information sources (e.g. databases, registers) used to identify studies and the date when each was last searched.	Yes
Risk of bias	5	Specify the methods used to assess risk of bias in the included studies.	Yes
Synthesis of results	6	Specify the methods used to present and synthesise results.	Yes
**RESULTS**			
Included studies	7	Give the total number of included studies and participants and summarise relevant characteristics of studies.	Yes
Synthesis of results	8	Present results for main outcomes, preferably indicating the number of included studies and participants for each. If meta-analysis was done, report the summary estimate and confidence/credible interval. If comparing groups, indicate the direction of the effect (i.e. which group is favoured).	Yes
**DISCUSSION**			
Limitations of evidence	9	Provide a brief summary of the limitations of the evidence included in the review (e.g. study risk of bias, inconsistency and imprecision).	Yes
Interpretation	10	Provide a general interpretation of the results and important implications.	Yes
**OTHER**			
Funding	11	Specify the primary source of funding for the review.	No, in full report
Registration	12	Provide the register name and registration number.	No, in full report

Table S3: Study variables

Dependent variables	Independent variables
Growth outcomes from discharge through six- and 12-months CA:<br>• Achieved weight (g), length (cm), HC (cm),<br>• Weight-, length-, and HC- for-age z-scores<br>• Change in weight-, length-, and HC-for-age<br><br>HM intake from discharge through six- and 12-months CA:<br>• Proportion of study population consuming HM<br>• Proportion of daily feedings volume as HM<br>• Duration of HM intake in weeks from discharge	• Gestational age at birth<br>• Sex<br>• Birth and discharge anthropometrics (weight (g), length (cm), HC (cm), and z-score for age)<br>• Route of HM intake (direct BF or expressed HM)<br>• Type of feeding at discharge (fortified HM, direct BF with PDF bottle feeds, unfortified HM, or direct BF with standard term formula bottle feeds)

Abbreviations: BF, breastfeeding; CA, corrected age; cm, centimeters; HC, head circumference; HM, human milk; g, gram

Table S4: Final search terms and MeSH terms used by database

**CINAHL (EBSCO)**	
#1	"Fortified Human Milk" OR "Fortified Breast Milk" OR "Fortified Human Milk" OR "Fortification of Human Milk" OR "Fortification of Human Milk" OR "Human Milk Fortif*" OR "Fortification of Breast Milk" OR "Breast Milk fortified" OR "Breast Milk fortification" OR "Breast Milk fortif*" OR Fortif* OR "multi-nutrient fortifi*" OR "Fortified Milk" OR "Fortified feed*" OR "Fortified Food*" OR "Supplemented Food*" OR "Enriched Food*" OR "Enriched Formula*" OR "Human Milk" OR "Milk Enrich*" OR "Breast Milk" OR "Breast Feeding" OR Breastfeeding OR "Breast Fed" OR "Preterm Discharge Formula" OR "Transition* Formula*" OR "Post-discharge Formula*" OR "Discharge Formula*" OR "Enriched Formula*" OR "Nutrient-enriched Formula*" OR "Infant Formula*" OR "Baby Formula" OR "Infant Nutrition*"
#2	"After Hospital Discharge" OR Post-discharge OR Postdischarge OR Discharge OR "After Discharge" OR "Following Discharge" OR "Subsequent Discharge" OR "Patient Discharge Summaries" OR "Patient Discharge" OR "Post discharge Care" OR "Discharge Summary" OR Aftercare OR "After-Care" OR "Follow-up Care" OR "After Release" OR Post-release
#3	"Premature Infant*" OR "Preterm Infant*" OR "Neonatal Prematurity" OR "Extremely Premature Infant" OR "Extremely Preterm Infant*" OR "Premature Birth*" OR "Preterm Birth*" OR "Very Low Birth Weight Infant" OR "Very Low Birth Weight" OR VLBW OR "Extremely Low Birth Weight Infant*" OR "Extremely Low Birth Weight" OR ELBW OR Neonate* OR Preterm* OR Premature*
#4	#1 AND #2 AND #3
**Cochrane Library (Wiley)**	
#1	("Fortified Human Milk"):ti,ab,kw
#2	("Fortified Breast Milk"):ti,ab,kw
#3	("Fortification of Human Milk"):ti,ab,kw
#4	(Fortification near/3 Human near/3 Milk):ti,ab,kw
#5	(Human NEXT Milk NEXT Fortif*):ti,ab,kw
#6	Human near/3 Milk near/3 Fortif*
#7	("Fortification of Breast Milk"):ti,ab,kw
#8	Fortification near/3 Breast near/3 Milk
#9	("Breast Milk fortified"):ti,ab,kw
#10	Breast near/3 Milk near/3 fortified
#11	("Breast Milk fortification"):ti,ab,kw
#12	(Breast near/3 Milk near/3 fortification):ti,ab,kw
#13	(Fortif*):ti
#14	(Fortif*):ab
#15	(multi-nutrient near/3 fortification):ti,ab,kw
#16	("fortified milk"):ti,ab,kw
#17	("Fortified feed"):ti,ab,kw
#18	(Fortified near/3 feed*):ti,ab,kw
#19	MeSH descriptor: [Food, Fortified] explode all trees
#20	("Fortified Food"):ti,ab,kw
#21	("Supplemented Food"):ti,ab,kw
#22	("Enriched Food"):ti,ab,kw
#23	("Enriched Formula"):ti,ab,kw
#24	MeSH descriptor: [Milk, Human] explode all trees
#25	("Human milk"):ti,ab,kw
#26	(Milk near/3 Enrich*):ti,ab,kw
#27	("breast milk"):ti,ab,kw
#28	MeSH descriptor: [Breast Feeding] explode all trees
#29	("Breast Feeding"):ti,ab,kw
#30	(Breastfeeding):ti,ab,kw

#31	("Breast Fed"):ti,ab,kw
#32	("Preterm Discharge Formula"):ti,ab,kw
#33	(Preterm near/3 Discharge near/3 Formula):ti,ab,kw
#34	("Transition Formula"):ti,ab,kw
#35	(Transition near/3 Formula):ti,ab,kw
#36	("Post-discharge Formula"):ti,ab,kw
#37	(post-discharge near/3 formula):ti,ab,kw
#38	("Discharge Formula"):ti,ab,kw
#39	("Enriched Formula"):ti,ab,kw
#40	("Nutrient-enriched Formula"):ti,ab,kw
#41	(Nutrient-enriched near/3 Formula):ti,ab,kw
#42	MeSH descriptor: [Infant Formula] explode all trees
#43	("Infant Formula"):ti,ab,kw
#44	("Baby Formula"):ti,ab,kw
#45	MeSH descriptor: [Infant Nutrition Disorders] explode all trees
#46	("Infant Nutrition"):ti,ab,kw
#47	#1 OR #2 OR #3 OR #4 OR #5 OR #6 OR #7 OR #8 OR #9 OR #10 OR #11 OR #12 OR #13 OR #14 OR #15 OR #16 OR #17 OR #18 OR #19 OR #20 OR #21 OR #22 OR #23 OR #24 OR #25 OR #26 OR #27 OR #28 OR #29 OR #30 OR #31 OR #32 OR #33 OR #34 OR #35 OR #36 OR #37 OR #38 OR #39 OR #40 OR #41 OR #42 OR #43 OR #44 OR #45 OR #46
#48	("After Hospital Discharge"):ti,ab,kw
#49	(After near/3 Hospital near/3 Discharge):ti,ab,kw
#50	("post-discharge"):ti,ab,kw
#51	("postdischarge"):ti,ab,kw
#52	(Discharge):ti
#53	(Discharge):ab
#54	("After Discharge"):ti,ab,kw
#55	("Following Discharge"):ti,ab,kw
#56	(Following near/3 Discharge):ti,ab,kw
#57	("Subsequent Discharge"):ti,ab,kw
#58	(Subsequent near/3 Discharge):ti,ab,kw
#59	MeSH descriptor: [Patient Discharge Summaries] explode all trees
#60	(Patient near/3 Discharge near/3 Summar*):ti,ab,kw
#61	("Patient Discharge"):ti,ab,kw
#62	("Post discharge Care"):ti,ab,kw
#63	(Post near/3 discharge near/3 Care):ti,ab,kw
#64	("Discharge Summary"):ti,ab,kw
#65	(Discharge near/3 Summary):ti,ab,kw
#66	MeSH descriptor: [Aftercare] explode all trees
#67	("Aftercare"):ti,ab,kw
#68	("After-Care"):ti,ab,kw
#69	("Follow-up Care"):ti,ab,kw
#70	("After Release"):ti,ab,kw
#71	("post-release"):ti,ab,kw
#72	#48 OR #49 OR #50 OR #51 OR #52 OR #53 OR #54 OR #55 OR #56 OR #57 OR #58 OR #59 OR #60 OR #61 OR #62 OR #63 OR #64 OR #65 OR #66 OR #67 OR #68 OR #69 OR #70 OR #71
#73	MeSH descriptor: [Infant, Premature] explode all trees
#74	("premature infant"):ti,ab,kw
#75	("preterm infant"):ti,ab,kw

#76	("Neonatal Prematurity"):ti,ab,kw
#77	(Neonat* near/3 Prematur*):ti,ab,kw
#78	MeSH descriptor: [Infant, Extremely Premature] explode all trees
#79	("Extremely Premature Infant"):ti,ab,kw
#80	(Extremely near/3 Premature near/3 Infant):ti,ab,kw
#81	("Extremely Preterm Infant"):ti,ab,kw
#82	(Extremely near/3 Preterm near/3 Infant):ti,ab,kw
#83	MeSH descriptor: [Premature Birth] explode all trees
#84	("premature birth"):ti,ab,kw
#85	("preterm birth"):ti,ab,kw
#86	MeSH descriptor: [Infant, Very Low Birth Weight] explode all trees
#87	("very low birth weight infant"):ti,ab,kw
#88	(Very near/3 Low near/3 Birth near/3 Weight near/3 Infant):ti,ab,kw
#89	("Very Low Birth Weight"):ti,ab,kw
#90	("VLBW infant"):ti,ab,kw
#91	(VLBW):ti,ab,kw
#92	MeSH descriptor: [Infant, Extremely Low Birth Weight] explode all trees
#93	("Extremely Low Birth Weight Infant"):ti,ab,kw
#94	(Extremely near/3 Low near/3 Birth near/3 Weight near/3 Infant):ti,ab,kw
#95	("Extremely Low Birth Weight"):ti,ab,kw
#96	(ELBW):ti,ab,kw
#97	(Neonate):ti,ab,kw
#98	("preterm"):ti,ab,kw
#99	("premature"):ab
#100	#73 OR #74 OR #75 OR #76 OR #77 OR #78 OR #79 OR #80 OR #81 OR #82 OR #83 OR #84 OR #85 OR #86 OR #87 OR #88 OR #89 OR 90 OR #91 OR #92 OR #93 OR #94 OR #95 OR #96 OR #97 OR #98 OR #99
#101	#47 AND #72 AND #100
#1	("Fortified Human Milk"):ti,ab,kw
#2	("Fortified Breast Milk"):ti,ab,kw
#3	("Fortification of Human Milk"):ti,ab,kw
#4	(Fortification near/3 Human near/3 Milk):ti,ab,kw
#5	(Human NEXT Milk NEXT Fortif*):ti,ab,kw
#6	Human near/3 Milk near/3 Fortif*
#7	("Fortification of Breast Milk"):ti,ab,kw
#8	Fortification near/3 Breast near/3 Milk
#9	("Breast Milk fortified"):ti,ab,kw
#10	Breast near/3 Milk near/3 fortified
#11	("Breast Milk fortification"):ti,ab,kw
#12	(Breast near/3 Milk near/3 fortification):ti,ab,kw
#13	(Fortif*):ti
#14	(Fortif*):ab
#15	(multi-nutrient near/3 fortification):ti,ab,kw
#16	("fortified milk"):ti,ab,kw
#17	("Fortified feed"):ti,ab,kw
#18	(Fortified near/3 feed*):ti,ab,kw
#19	MeSH descriptor: [Food, Fortified] explode all trees
#20	("Fortified Food"):ti,ab,kw
#21	("Supplemented Food"):ti,ab,kw
#22	("Enriched Food"):ti,ab,kw

#23	("Enriched Formula"):ti,ab,kw
#24	MeSH descriptor: [Milk, Human] explode all trees
#25	("Human milk"):ti,ab,kw
#26	(Milk near/3 Enrich*):ti,ab,kw
#27	("breast milk"):ti,ab,kw
#28	MeSH descriptor: [Breast Feeding] explode all trees
#29	("Breast Feeding"):ti,ab,kw
#30	(Breastfeeding):ti,ab,kw
#31	("Breast Fed"):ti,ab,kw
#32	("Preterm Discharge Formula"):ti,ab,kw
#33	(Preterm near/3 Discharge near/3 Formula):ti,ab,kw
#34	("Transition Formula"):ti,ab,kw
#35	(Transition near/3 Formula):ti,ab,kw
#36	("Post-discharge Formula"):ti,ab,kw
#37	(post-discharge near/3 formula):ti,ab,kw
#38	("Discharge Formula"):ti,ab,kw
#39	("Enriched Formula"):ti,ab,kw
#40	("Nutrient-enriched Formula"):ti,ab,kw
#41	(Nutrient-enriched near/3 Formula):ti,ab,kw
#42	MeSH descriptor: [Infant Formula] explode all trees
#43	("Infant Formula"):ti,ab,kw
#44	("Baby Formula"):ti,ab,kw
#45	MeSH descriptor: [Infant Nutrition Disorders] explode all trees
#46	("Infant Nutrition"):ti,ab,kw
#47	#1 OR #2 OR #3 OR #4 OR #5 OR #6 OR #7 OR #8 OR #9 OR #10 OR #11 OR #12 OR #13 OR #14 OR #15 OR #16 OR #17 OR #18 OR #19 OR #20 OR #21 OR #22 OR #23 OR #24 OR #25 OR #26 OR #27 OR #28 OR #29 OR #30 OR #31 OR #32 OR #33 OR #34 OR #35 OR #36 OR #37 OR #38 OR #39 OR #40 OR #41 OR #42 OR #43 OR #44 OR #45 OR #46
#48	("After Hospital Discharge"):ti,ab,kw
#49	(After near/3 Hospital near/3 Discharge):ti,ab,kw
#50	("post-discharge"):ti,ab,kw
#51	("postdischarge"):ti,ab,kw
#52	(Discharge):ti
#53	(Discharge):ab
#54	("After Discharge"):ti,ab,kw
#55	("Following Discharge"):ti,ab,kw
#56	(Following near/3 Discharge):ti,ab,kw
#57	("Subsequent Discharge"):ti,ab,kw
#58	(Subsequent near/3 Discharge):ti,ab,kw
#59	MeSH descriptor: [Patient Discharge Summaries] explode all trees
#60	(Patient near/3 Discharge near/3 Summar*):ti,ab,kw
#61	("Patient Discharge"):ti,ab,kw
#62	("Post discharge Care"):ti,ab,kw
#63	(Post near/3 discharge near/3 Care):ti,ab,kw
#64	("Discharge Summary"):ti,ab,kw
#65	(Discharge near/3 Summary):ti,ab,kw
#66	MeSH descriptor: [Aftercare] explode all trees
#67	("Aftercare"):ti,ab,kw
#68	("After-Care"):ti,ab,kw

#69	("Follow-up Care"):ti,ab,kw
#70	("After Release"):ti,ab,kw
#71	("post-release"):ti,ab,kw
#72	#48 OR #49 OR #50 OR #51 OR #52 OR #53 OR #54 OR #55 OR #56 OR #57 OR #58 OR #59 OR #60 OR #61 OR #62 OR #63 OR #64 OR #65 OR #66 OR #67 OR #68 OR #69 OR #70 OR #71
#73	MeSH descriptor: [Infant, Premature] explode all trees
#74	("premature infant"):ti,ab,kw
#75	("preterm infant"):ti,ab,kw
#76	("Neonatal Prematurity"):ti,ab,kw
#77	(Neonat* near/3 Prematur*):ti,ab,kw
#78	MeSH descriptor: [Infant, Extremely Premature] explode all trees
#79	("Extremely Premature Infant"):ti,ab,kw
#80	(Extremely near/3 Premature near/3 Infant):ti,ab,kw
#81	("Extremely Preterm Infant"):ti,ab,kw
#82	(Extremely near/3 Preterm near/3 Infant):ti,ab,kw
#83	MeSH descriptor: [Premature Birth] explode all trees
#84	("premature birth"):ti,ab,kw
#85	("preterm birth"):ti,ab,kw
#86	MeSH descriptor: [Infant, Very Low Birth Weight] explode all trees
#87	("very low birth weight infant"):ti,ab,kw
#88	(Very near/3 Low near/3 Birth near/3 Weight near/3 Infant):ti,ab,kw
#89	("Very Low Birth Weight"):ti,ab,kw
#90	("VLBW infant"):ti,ab,kw
#91	(VLBW):ti,ab,kw
#92	MeSH descriptor: [Infant, Extremely Low Birth Weight] explode all trees
#93	("Extremely Low Birth Weight Infant"):ti,ab,kw
#94	(Extremely near/3 Low near/3 Birth near/3 Weight near/3 Infant):ti,ab,kw
#95	("Extremely Low Birth Weight"):ti,ab,kw
#96	(ELBW):ti,ab,kw
#97	(Neonate):ti,ab,kw
#98	("preterm"):ti,ab,kw
#99	("premature"):ab
#100	#73 OR #74 OR #75 OR #76 OR #77 OR #78 OR #79 OR #80 OR #81 OR #82 OR #83 OR #84 OR #85 OR #86 OR #87 OR #88 OR #89 OR 90 OR #91 OR #92 OR #93 OR #94 OR #95 OR #96 OR #97 OR #98 OR #99
#101	#47 AND #72 AND #100
**Dissertation & Thesis Global (ProQuest)**	
#1	ANYWHERE<br>"Fortified Human Milk" OR "Fortified Breast Milk" OR "Fortified Human Milk" OR "Fortification of Human Milk" OR "Fortification of Human Milk" OR "Human Milk Fortif*" OR "Fortification of Breast Milk" OR "Breast Milk fortified" OR "Breast Milk fortification" OR "Breast Milk fortif*" OR Fortif* OR "multi-nutrient fortifi*" OR "Fortified Milk" OR "Fortified feed*" OR "Fortified Food*" OR "Supplemented Food*" OR "Enriched Food*" OR "Enriched Formula*" OR "Human Milk" OR "Milk Enrich*" OR "Breast Milk" OR "Breast Feeding" OR Breastfeeding OR "Breast Fed" OR "Preterm Discharge Formula" OR "Transition* Formula*" OR "Post-discharge Formula*" OR "Discharge Formula*" OR "Enriched Formula*" OR "Nutrient-enriched Formula*" OR "Infant Formula*" OR "Baby Formula" OR "Infant Nutrition*"
#2	ABSTRACT<br>"After Hospital Discharge" OR Post-discharge OR Postdischarge OR Discharge OR "After Discharge" OR "Following Discharge" OR "Subsequent Discharge" OR "Patient Discharge Summaries" OR "Patient Discharge" OR "Post discharge Care" OR "Discharge Summary" OR Aftercare OR "After-Care" OR "Follow-up Care" OR "After Release" OR Post-release

#3	ABSTRACT<br>"Premature Infant*" OR "Preterm Infant*" OR "Neonatal Prematurity" OR "Extremely Premature Infant" OR "Extremely Preterm Infant*" OR "Premature Birth*" OR "Preterm Birth*" OR "Very Low Birth Weight Infant" OR "Very Low Birth Weight" OR VLBW OR "Extremely Low Birth Weight Infant*" OR "Extremely Low Birth Weight" OR ELBW OR Neonate* OR Preterm* OR Premature
#4	#1 AND #2 AND #3
**Embase (Elsevier)**	
#1	('fortified human milk' OR 'fortified breast milk' OR (fortified NEAR/3 human NEAR/3 milk) OR (fortified NEAR/3 breast NEAR/3 milk) OR 'fortification of human milk' OR (fortification NEAR/3 human NEAR/3 milk) OR (human NEAR/3 milk NEAR/3 fortif*) OR 'fortification of breast milk' OR (fortification NEAR/3 breast NEAR/3 milk) OR 'breast milk fortified' OR (breast NEAR/3 milk NEAR/3 fortified) OR 'breast milk fortification' OR (breast NEAR/3 milk NEAR/3 fortification) OR 'breast milk fortif*' OR (breast NEAR/3 milk NEAR/3 fortif*) OR 'fortif*':ab,ti OR 'multi-nutrient fortifi*' OR ('multi nutrient' NEAR/3 fortifi*) OR 'fortified milk' OR 'fortified feed' OR 'fortified food'/exp OR 'fortified food' OR 'supplemented food*' OR 'enriched food*' OR 'enriched formula' OR 'human milk' OR 'milk enrich*' OR 'breast milk'/exp OR 'breast feeding'/exp OR 'breast feeding' OR 'breastfeeding' OR 'breast fed' OR (preterm NEAR/3 discharge NEAR/3 formula) OR 'transition* formula*' OR (transition* NEAR/3 formula*) OR 'post-discharge formula*' OR ('post discharge' NEAR/3 formula*) OR 'discharge formula' OR (discharge NEAR/3 formula) OR 'enriched formula*' OR (enrich* NEAR/3 formula) OR 'nutrient-enriched formula' OR ('nutrient enriched' NEAR/3 formula) OR 'infant formula' OR 'artificial milk'/exp OR 'baby formula' OR 'baby food'/exp OR 'infant nutrition' OR 'infant nutrition'/de)
#2	('after hospital discharge' OR (after NEAR/3 hospital NEAR/3 discharge) OR 'post discharge' OR 'postdischarge' OR 'discharge':ti,ab OR 'following discharge' OR (following NEAR/3 discharge) OR 'subsequent discharge' OR (subsequent NEAR/3 discharge) OR 'patient discharge summaries' OR (patient NEAR/3 discharge NEAR/3 summar*) OR 'patient discharge' OR 'post-discharge care' OR 'discharge summary' OR 'aftercare'/de OR 'aftercare':ti,ab OR 'after-care':ti,ab OR 'follow-up care' OR 'after release' OR 'post-release')
#3	('premature infant' OR 'prematurity'/exp OR 'preterm infant*' OR 'neonatal prematurity' OR (neonat* NEAR/3 prematur*) OR (neonat* NEAR/3 premterm*) OR (neonat* NEAR/3 preterm*) OR 'extremely premature birth'/exp OR 'extremely premature birth' OR 'extremely premature infant*' OR 'extremely preterm infant*' OR (extremely NEAR/3 preterm NEAR/3 infant*) OR (extremely NEAR/3 preterm NEAR/3 birth) OR 'premature birth' OR 'preterm birth' OR 'very low birth weight'/exp OR 'very low birth weight' OR vlbw OR (extremely NEAR/3 low NEAR/3 birth NEAR/3 weight) OR elbw OR 'neonate*':ti,ab OR 'preterm*':ti,ab OR 'prematur*':ti,ab)
#4	[english]/lim
#5	#1 AND #2 AND #3 AND #4
**PubMed (NLM)**	
#1	("Fortified Human Milk" OR "Fortified Breast Milk" OR "Fortified Human Milk"[Title/Abstract:~3] OR "Fortification of Human Milk" OR "Fortification of Human Milk"[Title/Abstract:~3] OR "Human Milk Fortif*" OR "Fortification of Breast Milk"[Title/Abstract:~3] OR "Breast Milk fortified"[Title/Abstract:~3] OR "Breast Milk fortification"[Title/Abstract:~3] OR "Breast Milk fortif*" OR Fortif*[tiab] OR "multi-nutrient fortifi*" OR "Fortified Milk" OR "Fortified feed*" OR "Food, Fortified"[Mesh] OR "Fortified Food*" OR "Supplemented Food*" OR "Enriched Food*" OR "Enriched Formula*" OR "Milk, Human"[Mesh] OR "Human Milk" OR "Milk Enrich*" OR "Breast Milk" OR "Breast Feeding"[Mesh] OR "Breast Feeding" OR Breastfeeding OR "Breast Fed" OR "Preterm Discharge Formula"[Title/Abstract:~5] OR "Transition* Formula*" OR "Post-discharge Formula*" OR "Discharge Formula*" OR "Enriched Formula*" OR "Nutrient-enriched Formula*" OR "Infant Formula"[Mesh] OR "Infant Formula*" OR "Baby Formula" OR "Infant Nutrition Disorders"[Mesh] OR "Infant Nutrition*")
#2	("After Hospital Discharge"[Title/Abstract:~5] OR Post-discharge OR Postdischarge OR Discharge OR "After Discharge" OR "Following Discharge"[Title/Abstract:~5] OR "Subsequent Discharge"[Title/Abstract:~5] OR "Patient Discharge Summaries"[Mesh] OR "Patient Discharge" OR "Post discharge Care" OR "Discharge Summary" OR "Aftercare"[Mesh] OR Aftercare[tiab] OR "After-Care"[tiab] OR "Follow-up Care" OR "After Release" OR Post-release)
#3	("Infant, Premature"[Mesh] OR "Premature Infant*" OR "Preterm Infant*" OR "Neonatal Prematurity" OR "Infant, Extremely Premature"[Mesh] OR "Extremely Premature Infant" OR "Extremely Preterm Infant*" OR "Premature Birth"[Mesh] OR "Premature Birth*" OR "Preterm Birth*" OR "Infant, Very Low Birth Weight"[Mesh] OR "Very Low Birth Weight Infant" OR

	"Very Low Birth Weight" OR VLBW OR "Infant, Extremely Low Birth Weight"[Mesh] OR "Extremely Low Birth Weight Infant*" OR "Extremely Low Birth Weight" OR ELBW OR Neonate*[tiab] OR Preterm*[tiab] OR Premature*[tiab])
#4	#1 AND #2 AND #3
**Scopus (Elsevier)**	
#1	ABSTRACT "Fortified Human Milk" OR "Fortified Breast Milk" OR "Fortified Human Milk" OR "Fortification of Human Milk" OR "Fortification of Human Milk" OR "Human Milk Fortif*" OR "Fortification of Breast Milk" OR "Breast Milk fortified" OR "Breast Milk fortification" OR "Breast Milk fortif*" OR Fortif* OR "multi-nutrient fortifi*" OR "Fortified Milk" OR "Fortified feed*" OR "Fortified Food*" OR "Supplemented Food*" OR "Enriched Food*" OR "Enriched Formula*" OR "Human Milk" OR "Milk Enrich*" OR "Breast Milk" OR "Breast Feeding" OR Breastfeeding OR "Breast Fed" OR "Preterm Discharge Formula" OR "Transition* Formula*" OR "Post-discharge Formula*" OR "Discharge Formula*" OR "Enriched Formula*" OR "Nutrient-enriched Formula*" OR "Infant Formula*" OR "Baby Formula" OR "Infant Nutrition*"
#2	TITLE-ABSTRACT-KEYWORDS "After Hospital Discharge" OR Post-discharge OR Postdischarge OR Discharge OR "After Discharge" OR "Following Discharge" OR "Subsequent Discharge" OR "Patient Discharge Summaries" OR "Patient Discharge" OR "Post discharge Care" OR "Discharge Summary" OR Aftercare OR "After-Care" OR "Follow-up Care" OR "After Release" OR Post-release
#3	TITLE-ABSTRACT-KEYWORDS "Premature Infant*" OR "Preterm Infant*" OR "Neonatal Prematurity" OR "Extremely Premature Infant" OR "Extremely Preterm Infant*" OR "Premature Birth*" OR "Preterm Birth*" OR "Very Low Birth Weight Infant" OR "Very Low Birth Weight" OR VLBW OR "Extremely Low Birth Weight Infant*" OR "Extremely Low Birth Weight" OR ELBW OR Neonate* OR Preterm* OR Premature*
#4	#1 AND #2 AND #3
**Web of Sciences (Clarivate)**	
#1	TOPIC "Fortified Human Milk" OR "Fortified Breast Milk" OR "Fortification of Human Milk" OR "Fortification of Human Milk" OR "Human Milk Fortif*" OR "Fortification of Breast Milk" OR "Breast Milk fortified" OR "Breast Milk fortification" OR "Breast Milk fortif*" OR Fortif* OR "multi-nutrient fortifi*" OR "Fortified Milk" OR "Fortified feed*" OR "Fortified Food*" OR "Supplemented Food*" OR "Enriched Food*" OR "Enriched Formula*" OR "Human Milk" OR "Milk Enrich*" OR "Breast Milk" OR "Breast Feeding" OR Breastfeeding OR "Breast Fed" OR "Preterm Discharge Formula" OR "Transition* Formula*" OR "Post-discharge Formula*" OR "Discharge Formula*" OR "Enriched Formula*" OR "Nutrient-enriched Formula*" OR "Infant Formula*" OR "Baby Formula" OR "Infant Nutrition*"
#2	TOPIC "After Hospital Discharge" OR Post-discharge OR Postdischarge OR Discharge OR "After Discharge" OR "Following Discharge" OR "Subsequent Discharge" OR "Patient Discharge Summaries" OR "Patient Discharge" OR "Post discharge Care" OR "Discharge Summary" OR Aftercare OR "After-Care" OR "Follow-up Care" OR "After Release" OR Post-release
#3	TOPIC "Premature Infant*" OR "Preterm Infant*" OR "Neonatal Prematurity" OR "Extremely Premature Infant" OR "Extremely Preterm Infant*" OR "Premature Birth*" OR "Preterm Birth*" OR "Very Low Birth Weight Infant" OR "Very Low Birth Weight" OR VLBW OR "Extremely Low Birth Weight Infant*" OR "Extremely Low Birth Weight" OR ELBW OR Neonate* OR Preterm* OR Premature*
#4	#1 AND #2 AND #3
**Google Scholar**	
#1	(Fortified OR Fortification OR Fortifier OR "Discharge Formula" OR "Enriched Formula")
#2	(Post-discharge OR Postdischarge OR Discharge OR Aftercare)
#3	(Premature OR Prematurity OR Preterm OR "Very Low Birth Weight" OR VLBW OR "Extremely Low Birth Weight" OR ELBW OR Neonate*)
#4	#1 AND #2 AND #3
